# Incomplete cell disruption of resistant microbes

**DOI:** 10.1038/s41598-019-42188-9

**Published:** 2019-04-04

**Authors:** Robert Starke, Nico Jehmlich, Trinidad Alfaro, Alice Dohnalkova, Petr Capek, Sheryl L. Bell, Kirsten S. Hofmockel

**Affiliations:** 1 Environmental Molecular Sciences Laboratory, Pacific Northwest National Laboratory, Richland, Washington USA; 2 Laboratory of Environmental Microbiology, Institute of Microbiology of the CAS, Praha, Czech Republic; 3 Helmholtz-Center for Environmental Research, UFZ, Leipzig, Germany; 4 Department of Ecology, Evolution and Organismal Biology, Iowa State University, Iowa, USA

## Abstract

Biomolecules for OMIC analysis of microbial communities are commonly extracted by bead-beating or ultra-sonication, but both showed varying yields. In addition to that, different disruption pressures are necessary to lyse bacteria and fungi. However, the disruption efficiency and yields comparing bead-beating and ultra-sonication of different biological material have not yet been demonstrated. Here, we show that ultra-sonication in a bath transfers three times more energy than bead-beating over 10 min. TEM imaging revealed intact gram-positive bacterial and fungal cells whereas the gram-negative bacterial cells were destroyed beyond recognition after 10 min of ultra-sonication. DNA extraction using 10 min of bead-beating revealed higher yields for fungi but the extraction efficiency was at least three-fold lower considering its larger genome. By our critical viewpoint, we encourage the review of the commonly used extraction techniques as we provide evidence for a potential underrepresentation of resistant microbes, particularly fungi, in ecological studies.

## Introduction

Finding efficient cell disruption techniques is crucial to various scientific fields. For example, biodiesel as a fuel alternative can be generated from algal lipids^1^. Algae are resistant to lysis due to a high cellulose content^2^, which is why harsh mechanical methods are needed to ensure complete lipid extraction. Such methods include autoclaving, bead-beating, high-pressure homogenization and ultra-sonication^3^, which were previously reported to result in varying lipid yields^4^. Similarly, increasing treatment time of different cell disruption techniques revealed increased protein yields from yeast^5^ with a logarithmic relationship between pressure and yield (R^2^ = 0.96). Together these results suggest an efficiency plateau for the extraction of proteins and lysis of cells (Fig. S1a). Ultra-sonication resulted in five-fold higher protein yields as compared to high pressure homogenization, and 20-fold higher protein yields as compared to hydrodynamic cavitation. A previous study demonstrated higher resistance in fungi and several gram-positive bacteria towards cell disruption than other gram-positive bacteria or gram-negative bacteria (Fig. S1b)^6^. In particular, gram-positive *Streptococcus faecalis* (now classified as *Enterococcus faecalis*) and *Staphylococcus aureus*, and fungal *Aspergillus fumigates* and *Fusarium* spp. were more resistant as compared to gram-positive *Bacillus subtilis* and *Lactobacillus casei*, and the gram-negative *Escherichia coli*. Differential resistance during cell disruption can bias the extraction of biomolecules towards easy-to-lyse microbes, which can skew the previously estimated microbiome compositions, ranging from the human gut^7^ to forest soil ecosystems^8^. These differences in resistance could derive from differences in the cell wall chemistry since muramic acid exclusively occurs in bacterial cell walls^9,10^ whereas fungal cell walls are the dominant source of glucosamine^9^ or from the varying degrees of cell aggregates in fungi to shield individual cells from the disruption treatment^11^.

Among commonly used cell disruption techniques, hydrodynamic cavitation (3,300 kJ in 10 min), high pressure homogenization (2,000 kJ in 10 min) and ultra-sonication (54 kJ in 10 min) transfer different quantities of energy to the cell^5^ but, more precisely, the energy is applied in a distinct volume. The resulting pressure, as energy per volume on the cell, increases from ultra-sonication in a bath (60 MPa) to high pressure homogenization (600 MPa) and ultra-sonication with a probe (60,000 MPa). Otherwise, the pressure from bead-beating in a liquid cannot be easily estimated by knowing the power of the instrument as it comprises complex fluid dynamics^12^. However, since the change in temperature can be correlated to energy input as heat in a closed system (Eq. 2), the pressures of bead-beating and ultra-sonication can be quantitatively compared when a similar thermodynamic system with identical volume is used.

**Figure 1:**
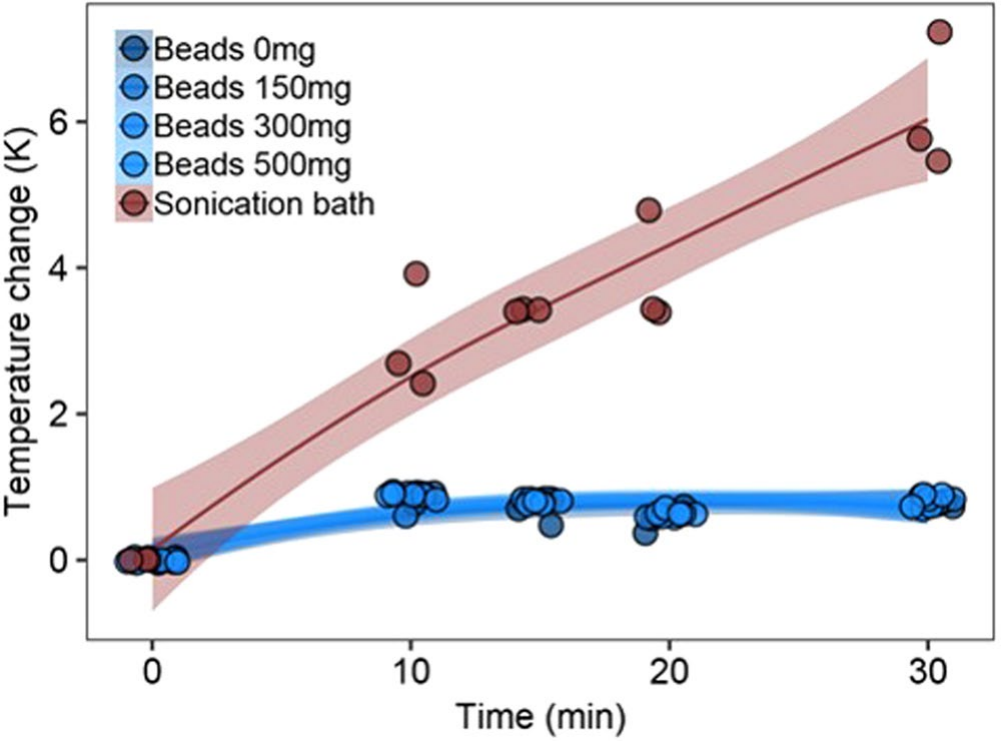
Temperature change of water (V = 2 ml) in a closed system incubated using bead-beating at 10 Hz with different amounts of beads (0, 150, 250 and 500 mg) and an ultra-sonication bath over 30 min (n = 3). Ultra-sonication showed a linear relation (R^2^ = 0.87) whereas logarithmic relations were found for bead-beating (R^2^_0mg_ = 0.77, R^2^_150mg_ = 0.86, R^2^_300mg_ = 0.83, R^2^_500mg_ = 0.85) which plateaued after 10 min regardless the amount of beads.

To quantitatively compare cell disruption techniques, we compared the heat transferred to 2 mL water in a closed tube with varying amounts of 0.1 mm glass beads, which decrease the total volume of water in tube, in an ultra-sonication bath or during bead-beating at 10 Hz for 10, 20 and 30 min. Without considering the air circulation, the heat capacities were within one magnitude of order given the isobaric volumetric heat capacity of water (4.1796 J cm^−3^ K^−1^) as surrounding medium during ultra-sonication in a bath (200 cm^3^) and the isobaric volumetric heat capacity of air (0.000121 J cm^−3^ K^−1^) as surrounding medium during bead-beating in a fume hood (250,000 cm^3^). Ultra-sonication in a bath (25.1 ± 6.6 J) resulted in three-fold higher heat transfers as compared to bead-beating (7.3 ± 0.5 J) after 10 min (Table 1). Using bead-beating, the transferred heat was similar over time and with increasing amount of beads, whereas the heat transferred by ultra-sonication linearly increased at a rate of 2 K per 10 min over 30 min (Fig. 1). In ultra-sonication, the applied pressure on the cell can directly be related to the power of the instrument and the volume in which the treatment is performed; energy is immediately transferred to the cells via hydrodynamic cavitation through sound waves, which affects chemical systems^13^ corresponding to acoustic cavitation in which bubbles are formed, grown, and imploded in a liquid^14^. On the contrary, the power of the instrument in bead-beating is used to shake the vessel containing the solution with beads and potentially the biological material. Most of the applied pressure is lost to overcome the inertia of the solution in accordance to Newton’s first law: “An object that is at rest will stay at rest unless a force acts upon it”. Once in motion, the beads lose energy at every collision whether that is with the desired cells or the undesired collision with other beads, non-biological particles or the surrounding vessel. Logically, the beads-to-cell ratio will determine the efficiency of the cell disruption while the increase in non-biological particles will increase the amount of undesired collisions, which could finally lower the yields. The combined effects of both inertia and undesired collisions during bead-beating lead to higher heat transfers during ultra-sonication.

**Table 1:** Heat (Q) transferred from bead-beating and ultra-sonication.

Method	Beads (mg)	Q_10min_ (J)	Q_20min_ (J)	Q_30min_ (J)
Ultra-sonication	0	25.1 ± 6.6	32.3 ± 6.8	51.6 ± 7.6
Bead-beating	0	7.3 ± 0.5	4.7 ± 1.3	6.7 ± 0.8
	150	6.2 ± 1.4	4.6 ± 0.1	6.2 ± 0.1
	300	6.3 ± 0.5	4.4 ± 0.1	5.3 ± 0.4
	500	5.8 ± 0.3	4.1 ± 0.3	6.2 ± 0.1

**Figure 2:**
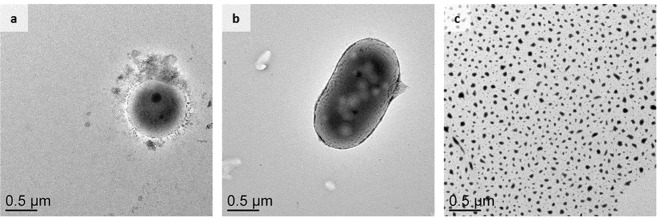
TEM images of representative cells of the Gram-positive bacteria (**a**) and the fungal enrichment culture (**b**) derived from KBS switchgrass soil after cell disruption with an ultra-sonication bath for 10 minutes. With identical treatment, no intact cells were found in the Gram-negative bacterial enrichment culture (**c**).

Noteworthy, ultra-sonication in a bath for 10 min revealed a strong resistance of the fungal and the gram-positive bacterial enrichment culture, both remained almost intact, whereas no intact cells could be found in the gram-negative bacterial enrichment culture after identical treatment, suggesting complete cell disruption beyond recognition (Figs 2 and S2–4). In fact, this could be the reason for the underrepresentation of resistant microbes, particularly fungi, in ecological studies^15–17^ that might skew microbiomes of various nature towards easy-to-lyse microbes. Even though we specifically searched for single cells, the gram-positive bacterial and the fungal enrichment cultures showed higher amounts of cell aggregation compared to the gram-negative bacterial enrichment culture. The latter had clearly separated single cells (Fig. S5), which cannot protect individual cells from disruption as it was previously reported^11^. To complement imaging after sonication, we extracted DNA with a standard kit using 10 min of bead-beating for cell disruption. Resulting DNA yields were comparable between the bacterial enrichment cultures with slightly higher yields for the fungal culture (Table 2). Given the variation in DNA content between bacterial, specifically the gram-positive *Bacillus*^18^ and the gram-negative *Bradyrhizobium*^19^ that were enriched here, and fungal cells, for instance *Aspergillus nidulans*^20^, we have direct evidence for incomplete disruption of fungi since their genome is larger^21^. Logically, one would expect DNA yields similar to the genome size if the fungal and gram-negative cells were equally lysed, which would equal to 0.0690 g/L in fungi. To our surprise, despite lower DNA content of the gram-positive bacteria, comparable yields between gram-negative and gram-positive cells imply that complete lysis might not be necessary for sufficient DNA extraction that, however, could be different when other biomolecules such as RNA or proteins are extracted. Moving forward, cell disruption efficiencies can be increased with increasing treatment time but, depending on the type of biomolecule and the organism of interest, denaturation processes have to be considered as high pressure cell disruption will result in a higher change in temperature of the carrier liquid. Concluding, TEM images showed that the commonly applied disruption techniques bead-beating and ultra-sonication over 10 min are not sufficient to disrupt different biological material completely and uniformly but DNA extraction from the same material only revealed a bias towards fungi whereas gram-positive bacteria despite incomplete lysis proportionally yielded more DNA than gram-negative bacteria. Similarly the extraction efficiency of other biomolecules such as RNA, proteins and intracellular metabolites from different biological material could not be impacted by incomplete cell disruption but this has to be validated. Based on the correlation of higher disruption pressure and higher protein yields from yeast, we suggest longer treatment times and/or the consideration of other disruption techniques that, however, could impact the quality of the extracted biomolecules. Aggregation as potential mechanism of resistance^11^ in cell cultures raises concerns about the disruption efficiencies in environmental samples, particularly in soil where aggregates, even though these result from different processes in the form of rearrangement, flocculation and cementation^22^, are crucial to protect from erosion^23^ and decomposition^24^, but could also provide protection against cell disruption. Even though we revealed differences in disruption efficiencies using bead-beating and ultra-sonication, our results have to be further validated, for instance by comparing the microbial community composition extracted with different techniques and treatment times, especially in environments with a high abundance of fungal and gram-positive bacterial biomass, and in soil samples with a high amount of non-biological particles that could negatively impact the disruption efficiency by protecting biological material via cell aggregation and increasing the amount of undesired collisions during bead-beating. Moving forward, cell disruption protocols have to be critically reviewed as we provide strong evidence that commonly used disruption pressures are not sufficient to entirely lyse biological material with different resistance. Similarly OMIC analyses of environmental microbiomes must be interpreted with caution, acknowledging the differential resistance to lysing among morphologically distinct microbes.

**Table 2:** DNA yields after bead-beating for 10 min compared to growth yields and genome sizes.

Enrichment	Culture	Biomass C (μmol/mL)	Growth yield	DNA yield (g/L)	Genome size
NUA	Gram+	366.66	0.46	0.0218	4.2 Mbp^19^
YMA	Gram−	245.33	0.31	0.0230	9.1 Mbp^20^
CDA	Fungi	504.04	0.63	0.0291	30.0 Mbp^21^

## Materials and Methods

### Calculation of disruption pressures and energy output from bead-beating and ultra-sonication

The disruption pressures (*p*) of homogenization and ultra-sonication were determined by calculating the disruption energies, which equals the power of the instrument (*P* = 200 W) and the incubation time (*t* = 10 min) divided by the volume of the bath (*V* = 2,000 ml) or the liquid surrounding the probe (*V* = 2 ml) (Eq. 1).

1 $$P=\frac{P\ast t}{V}(1)$$

To validate the heat transfer, sterile ultra-pure water was mixed with 0.1 mm glass beads in varying amounts (0, 150, 300 and 500 mg) in triplicates to a total volume of 2 mL in an Eppendorf tube. Bead-beating (Mixer Mill MM 400, Retsch, Haan, Germany) and ultra-sonication in a bath (CPX 2800, Branson Ultrasonic SA, Carouge, Switzerland) were performed over 30 min. Water temperature in the tube was measured in the beginning and every 10 min for each treatment. The change in temperature was correlated to energy input as heat using Eq. 2 where *m* is the mass of water, which decreased with increasing amounts of glass beads in the liquid phase, and *c* the specific heat of the water.

2 $$Q=m\ast c\ast \Delta T$$

### Soil sampling, TEM imaging and DNA extraction

Soils originated from the Great Lakes Bioenergy Research Center (GLBRC) located at the W.K. Kellogg Biological Station (KBS) Long-Term Ecological Research Site (Hickory Corners, MI, 42°24′N, 85°24′W). No-till soil samples under perennial switchgrass (*Panicum virgatum*) were collected from 0–15 cm depth over three days in May 2017. The upper 5 cm were discarded to reduce the influence of surface litter on the soil. Bulk soil was transported to the lab on ice packs, and passed through a 2 mm sieve. Phylogenetically-specific agar was used to enrich gram-negative and gram-positive bacteria, and fungi from whole soil using Yeast mannitol agar (YMA)^25^ to enrich the gram-negatives *Bradyrhizobium* and *Rhodopseudomonas*, Nutrient agar (NUA)^26^ for the gram-positive *Bacillus*, and Czapeck Dox agar (CDA)^27^ as synthetic media for fungal growth. After two weeks of incubating soil on the phylogenetically-specific agar, grown colonies were transferred to broth comprising of 50 mL M9 mineral medium^28^ and incubated at 21 °C. Once the stationary phase was reached, the cell solution was centrifuged at 10,000 rpm for 15 min (Eppendorf Centrifuge 5810 R, Eppendorf North America, New York, USA). For TEM imaging, 50 μg of each cell pellet was resuspended in 2 mL sterile ultra-pure water and used to image intact cells or disrupted using an ultra-sonication bath for 10 min (Branson 2800 CPX, Branson Ultrasonics, Danbury, USA) to image lysed cells. A whole mount approach was applied for imaging of the cells. For each sample, a 5 μL drop of the cell solution was applied to a 100-mesh Cu grid covered with formvar support film sputtered with carbon (Electron Microscopy Sciences, Hatfield, PA, USA). The material was allowed to adhere to the grid for 1 min before the liquid was gently blotted with a filter paper, and the material was negatively stained with a 5 μL drop of Nano-W (Nanoprobes, Yaphank, NY, USA). After 3 s, the excess liquid was removed by wicking and the sample was allowed to air dry. Samples were examined with a Tecnai T-12 TEM (FEI) with a LaB6 filament operating at 120 kV. Images were collected digitally with a 2 × 2 K UltraScan CCD (Gatan, Pleasanton, CA, USA). For each sample, at least 50 regions within the grid containing multiple cells were analyzed before collecting representative images at 6,500x magnification. 50 μg of the remaining cell pellet was used for DNA extraction with the DNeasy PowerLyzer Microbial Kit part #12255-50 (Qiagen, Venlo, Netherlands).

## Supplementary information

Supplementary Information
