# Supplementary material for: Incomplete cell disruption of resistant microbes
Source: Sci Rep. 2019 Apr 4;9:5618. doi: 10.1038/s41598-019-42188-9 (PMC6449382; doi:10.1038/s41598-019-42188-9)
Supplement: Supplementary file 1 — Supplementary Information [file 41598_2019_42188_MOESM1_ESM.docx]

**Supplementary Information**

**Incomplete cell disruption of resistant microbes**

Robert Starke^1,2,*^, Nico Jehmlich^3^, Trinidad Alfaro^1^, Alice Dohnalkova^1^, Petr Capek^1^, Sheryl L. Bell^1^, Kirsten S. Hofmockel^1,4^

^1^Environmental Molecular Sciences Laboratory, Pacific Northwest National Laboratory, Richland, Washington, United States of America

^2^Laboratory of Environmental Microbiology, Institute of Microbiology of the CAS, Praha, Czech Republic

^3^Helmholtz-Center for Environmental Research, UFZ, Leipzig, Germany

^4^Department of Ecology, Evolution and Organismal Biology, Iowa State University

*Corresponding author: robert.starke@biomed.cas.cz (RS)

**Figures**

**
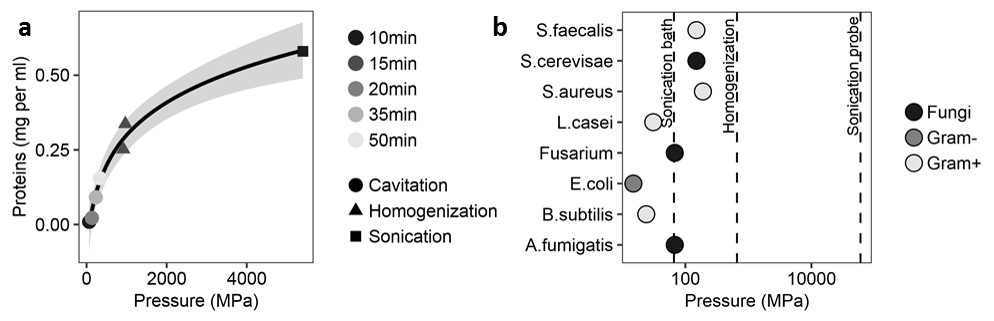
**

**Figure S1** The relationship of cell disruption pressure to extraction yield (a) and resistance (b). The resulting pressures from hydrodynamic cavitation, high pressure homogenization and ultra-sonication resulted in a logarithmic relation (R^2^=0.96) with the protein yield from yeast (a, data from Balasundaram & Pandit, 2001). The pressure to disrupt half of the cells varied between selected Gram-negative and Gram-positive bacteria, and fungi (b, data from Kelemen & Sharpe, 1979).


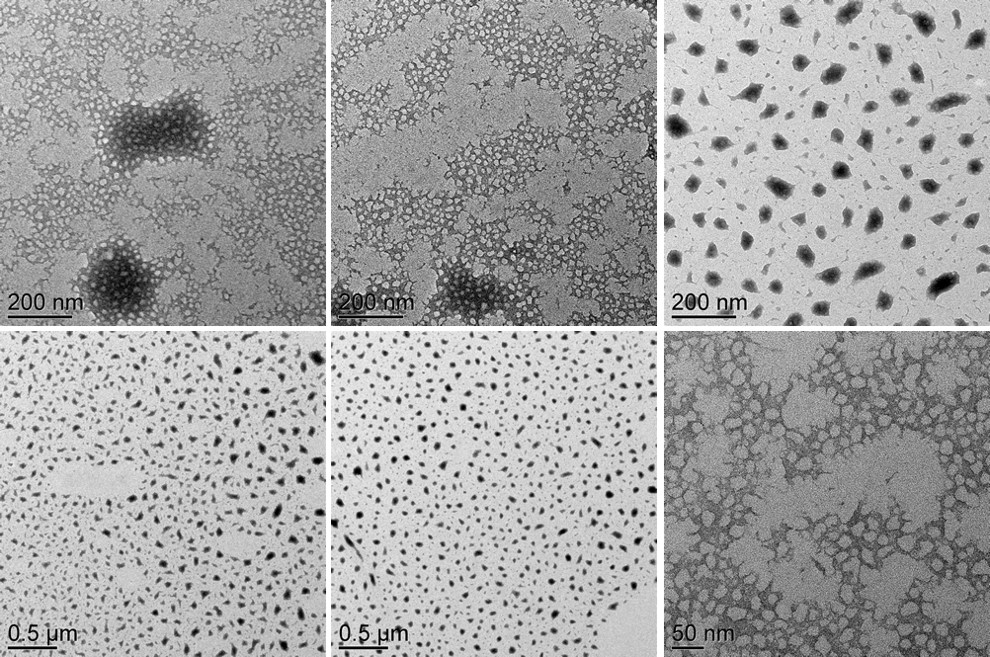


**Figure S2** A selection of TEM images from the Gram-negative bacterial enrichment culture from KBS switchgrass soil after cell disruption with an ultra-sonication bath for 10 minutes. No intact cells were detected. The magnification of the individual figures varied from 50 nm to 0.5 µm.


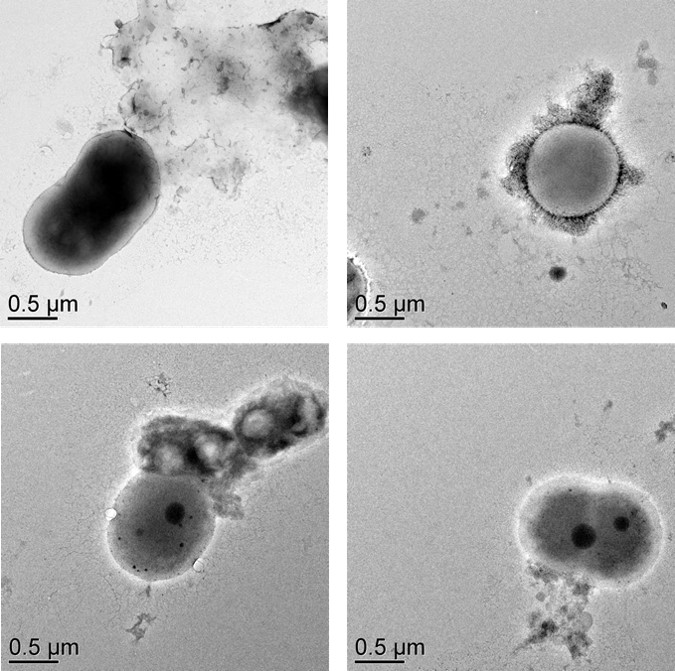


**Figure S3** A selection of TEM images from the Gram-positive bacterial enrichment culture from KBS switchgrass soil after cell disruption with an ultra-sonication bath for 10 minutes. Cells remain intact.


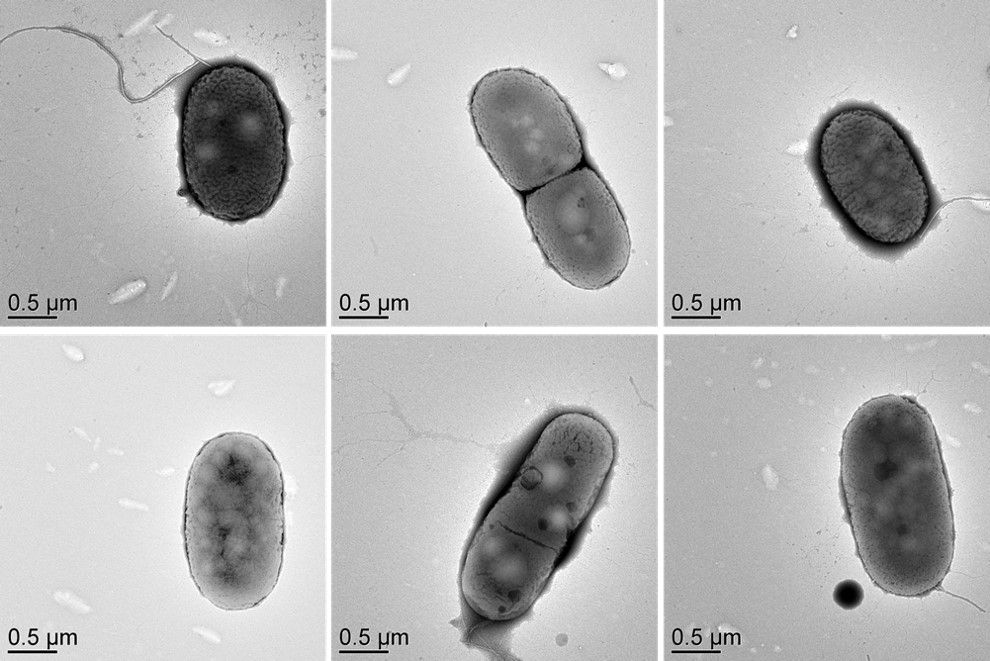


**Figure S4** A selection of TEM images from the fungal enrichment culture from KBS switchgrass soil after cell disruption with an ultra-sonication bath for 10 minutes. Cells remain intact.


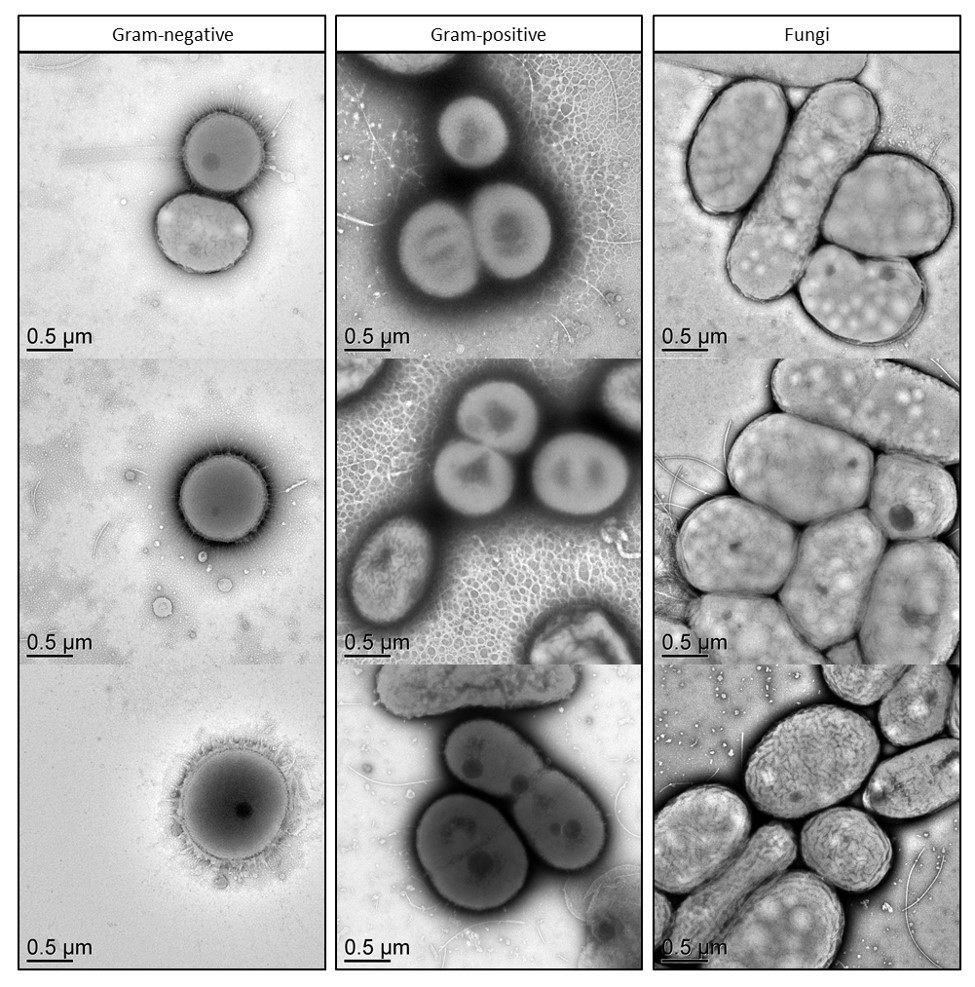


**Figure S5** TEM images of representative cells of the Gram-negative and the Gram-positive bacterial, and the fungal enrichment culture from Michigan whole soil grown under perennial switchgrass prior to cell disruption.
